# Solvothermal preparation of spherical Bi_2_O_3_ nanoparticles uniformly distributed on Ti_3_C_2_T_*x*_ for enhanced capacitive performance†

**DOI:** 10.1039/d1na00443c

**Published:** 2021-08-05

**Authors:** Tao Li, Xuefeng Chang, Lifang Mei, Xiayun Shu, Jidong Ma, Li Ouyang, Siyong Gu

**Affiliations:** Mechanical and Automotive Engineering, Xiamen University of Technology Xiamen Fujian 361024 P. R. China shuxiayun@xmut.edu.cn; Institute of Precision Actuation and Transmission Xiamen Fujian 361024 P. R. China; The Key Laboratory for Power Metallurgy Technology and Advanced Materials of Xiamen Xiamen Fujian 361024 P. R. China gu-siyong@163.com

## Abstract

Ti_3_C_2_T_*x*_ is a promising new two-dimensional layered material for supercapacitors with good electrical conductivity and chemical stability. However, Ti_3_C_2_T_*x*_ has problems such as collapse of the layered structure and low pseudocapacitance. In this paper, we propose Bi_2_O_3_–Ti_3_C_2_T_*x*_ nanocomposites prepared by a solvothermal method, study the impact of Bi_2_O_3_ loading on the phase state and microstructure, and evaluate the electrochemical performance of Bi_2_O_3_–Ti_3_C_2_T_*x*_. Studies have shown that spherical Bi_2_O_3_ particles were uniformly dispersed in the interlayer and surface of Ti_3_C_2_T_*x*_, which enlarged the interlayer spacing of the Ti_3_C_2_T_*x*_ and increased the pseudocapacitance. When the mass percentage of Bi_2_O_3_ and Ti_3_C_2_T_*x*_ was 30% (TB30), the specific capacity of TB30 was as high as 183 F g^−1^ at a current density of 0.2 A g^−1^, which was about 2.8 times that of Ti_3_C_2_T_*x*_ (TB0). Moreover, a typical asymmetric supercapacitor device assembled with TB0 as the positive electrode and TB30 as the negative electrode exhibited a high energy density of 3.92 W h kg^−1^ and a maximum power density of 36 000 W kg^−1^ and maintained 77.4% of the initial capacitance after 5000 cycles at a current density of 2 A g^−1^. Therefore, the Bi_2_O_3_–Ti_3_C_2_T_*x*_ as the negative electrode of supercapacitor has broad application prospects in the field of energy storage.

## Abstract 2

Ti_3_C_2_T_*x*_ is a promising new two-dimensional layered material for supercapacitors with good electrical conductivity and chemical stability.

## Graphical abstract

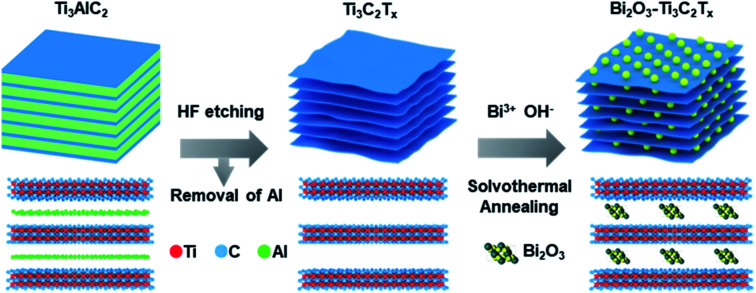

## 1. Introduction

With the rapid development of the global economy, fossil fuel consumption continues to increase, causing serious environmental pollution. The search for efficient, clean and sustainable renewable energy and energy storage technologies has become an important research area.^1^ At present, common electrochemical energy conversion and storage technologies include lithium-ion batteries, fuel cells, and supercapacitors. Supercapacitors have wide application potential because of their combined advantages of high power density, fast charging and discharging, long cycle lifetime and environmental friendliness.^2–4^

The performance of supercapacitors is mainly influenced by the electrolyte, separator, electrode materials and packaging technology, among which the electrode materials determine the energy density.^5,6^ Different electrode materials correspond to different energy storage mechanisms. Supercapacitors can be divided into electrochemical double-layer capacitors (EDLCs) and pseudocapacitors (PCs). EDLCs rely on the electrostatic interactions between the electrode and electrolyte to store charge, and PCs rely on the highly reversible redox reaction on the electrode surface to store energy.^7,8^ The ideal electrode material should have a large specific surface area, large interlayer spacing, excellent hydrophilicity, great stability, good conductivity and high pseudocapacitance.

Common electrode materials include carbon materials, metal oxide materials and conductive polymer materials.^9–13^ Ti_3_C_2_T_*x*_ is a new class of electrode material with high specific surface area, high hydrophilicity, good electrical conductivity and high chemical stability, with a layered structure similar to graphene, and has received widespread attention since its discovery in 2011.^14^ There are surface defects and porous structure after the corrosion of Ti_3_C_2_T_*x*_ by HF. The defects would be beneficial for increasing the contact area with the electrolyte. Therefore, the surface defects and porous structure provide large accessible surface area for cation intercalation^15^ and abundant active sites for the oxygen reduction reactions,^16^ facilitates easy charge-carrier transport, leading to enhanced electrochemical performance. These characteristics provide high electrode double-layer capacitance, pseudocapacitance, and reaction planes, making Ti_3_C_2_T_*x*_ suitable as an electrode material for supercapacitors. However, studies have found that the collapse of the layered structure and the small pseudocapacitance are the main factors limiting the performance of Ti_3_C_2_T_*x*_ electrode materials.^17,18^

An effective way to improve the performance is to introduce pseudocapacitive materials such as MnO_2_,^19^ TiO_2_,^20^ and polypyrrole^21^ into the Ti_3_C_2_T_*x*_ interlayer.^22^ Enlarging the interlayer spacing of Ti_3_C_2_T_*x*_ makes it conducive for a variety of ions, such as Na^+^, K^+^, NH_4_^+^, to reversibly enter the material to achieve charge storage and better pseudocapacitance.^12,28^ For instance, Yuan *et al.*^19^ successfully introduced MnO_2_, with good pseudocapacitive performance, into the Ti_3_C_2_T_*x*_ layers, which effectively prevented layer stacking and exhibited higher specific surface area than Ti_3_C_2_T_*x*_. The MnO_2_/Ti_3_C_2_T_*x*_ showed a specific capacitance of 254 F g^−1^ (0.5 A g^−1^). Zhu *et al.*^21^ studied a new way to enhance structural stability and interlayer spacing of Ti_3_C_2_T_*x*_ by forming a freestanding and conductive thin film through intercalating polypyrrole into layered Ti_3_C_2_T_*x*_ electrodes modified by polypyrrole. The results showed that due to the modification of polypyrrole, the surface capacitance of Ti_3_C_2_T_*x*_ reached 203 mF cm^−2^, and after long term cycling (20 000 cycles), there was no obvious capacity degradation.

Bismuth oxide (Bi_2_O_3_) is a pseudocapacitive material with excellent comprehensive performance owing to its high theoretical capacitance (1370 F g^−1^), suitable potential window, low cost and non-toxicity.^23^ Recent studies have shown that Bi_2_O_3_ can be used as an electrode material for supercapacitors. Furthermore, its electrochemical performance has been improved by combining with other electrode materials based on carbon and transition metal oxides, such as carbon nanotubes,^24^ graphene^25^ and MnO_2_.^26^ Thus, we expect that Bi_2_O_3_ would show an affinity for Ti_3_C_2_T_*x*_-based materials, which has not yet been reported in literature. In this work, we report a series of novel Bi_2_O_3_–Ti_3_C_2_T_*x*_ nanocomposites prepared by a facile solvothermal reaction. Bi_2_O_3_ was evenly loaded onto the interlayer and surface of Ti_3_C_2_T_*x*_, which successfully increased the interlayer spacing, stabilized the layered structure, and significantly improved the electrochemical performance. This work demonstrates that Bi_2_O_3_–Ti_3_C_2_T_*x*_ nanocomposites have an enhanced performance compared to pure Ti_3_C_2_T_*x*_

## 2. Experimental section

### 2.1 Fabrication procedures

#### Ti_3_C_2_T_*x*_ substrate

Initially, 5 g of Ti_3_AlC_2_ was slowly immersed in 60 mL of 40 wt% HF solution under magnetic stirring (700 rpm) for 24 h at room temperature. After cooling down to room temperature, the reaction products were washed several times with deionized water and absolute ethanol to pH >6. Finally, the products were dried at 60 °C for 12 h to obtain Ti_3_C_2_T_*x*_ matrix material.

#### Bi_2_O_3_–Ti_3_C_2_T_*x*_ nanocomposite

**Fig. 1:**
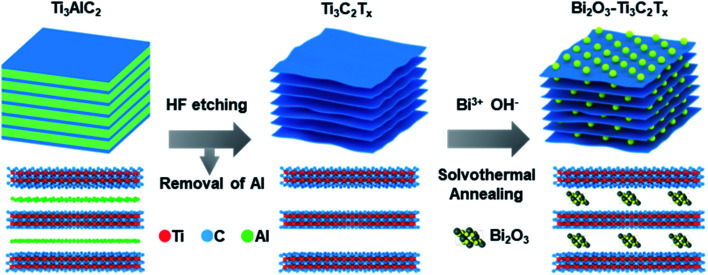
Schematic illustration of the synthesis route to the Bi_2_O_3_–Ti_3_C_2_T_*x*_ nanocomposites.

Bi(NO_3_)_3_·5H_2_O was used as the bismuth source, with ethylene glycol and ethanol as solvents and Ti_3_C_2_T_*x*_ as the substrate. Bi(NO_3_)_3_·5H_2_O and Ti_3_C_2_T_*x*_ were added into a mixed solution consisting of ethanol and ethylene glycol with 10 min of magnetic stirring and 30 min of ultrasonication to form a suspension. Then, the suspension was transferred into a 100 mL Teflon container and placed in a stainless steel autoclave for a solvothermal reaction at 160 °C for 5 h. After the reaction, the suspension was taken out, washed and dried to form a precursor. Finally, the precursor was put in a tube furnace for annealing at 300 °C for 2 h in N_2_ atmosphere to obtain the Bi_2_O_3_–Ti_3_C_2_T_*x*_ nanocomposite. By adjusting the ratio of Bi(NO_3_)_3_·5H_2_O to Ti_3_C_2_T_*x*_, Bi_2_O_3_–Ti_3_C_2_T_*x*_ nanocomposites with different Bi_2_O_3_ contents, *i.e.*, 0%, 10%, 20%, 30% and 40%, were prepared. The resulting samples are referred to as TB*n*, where T represents Ti_3_C_2_T_*x*_, B stands for Bi_2_O_3_, and *n* is the mass percentage of Bi_2_O_3_ and Ti_3_C_2_T_*x*_. Table 1 shows the proportion of raw materials used for preparing TB*n*. Fig. 1 schematically illustrates the preparation steps for the Bi_2_O_3_–Ti_3_C_2_T_*x*_ nanocomposites. The ionic interaction between negatively charged groups, such as –F, –O, and –OH functional groups at the surface and interlayer of Ti_3_C_2_T_*x*_ and positively charged Bi^3+^ cations, resulting in an even distribution of Bi^3+^ on Ti_3_C_2_T_*x*_ to form Bi^3+^–Ti_3_C_2_T_*x*_ intermediates.^41^ The formation of Bi_2_O_3_–Ti_3_C_2_T_*x*_ can be achieved during the solvothermal process. The uniform distribution of Bi_2_O_3_ at the surface and interlayers of Ti_3_C_2_T_*x*_ can effectively prevent layer stacking, significantly increase the contact area between Bi_2_O_3_ and Ti_3_C_2_T_*x*_, improve the electronic conductivity, and accelerate the rapid transfer of electrolyte ions, resulting in an increase in the electric double layer capacitance and intercalation pseudocapacitance.

**Table 1:** The proportion of raw materials used for preparing TB*n*

Sample	Ti_3_C_2_T_*x*_ (g)	Bi(NO_3_)_3_·5H_2_O (g)	(CH_2_OH)_2_ (mL)	C_2_H_6_O (mL)	Annotation
TB0	0.5	0	13	52	0%
TB10	0.5	0.12	13	52	10%
TB20	0.5	0.26	13	52	20%
TB30	0.5	0.45	13	52	30%
TB40	0.5	0.69	13	52	40%

### 2.2 Materials characterization

The crystal phases of the products were analyzed by X-ray diffraction (XRD) with Cu Kα radiation (Rigaku, D/max-RB12, Japan). X-ray photoelectron spectroscopy (XPS, PHI-5300) was used to investigate the chemical valence composition of the surface elements of the product. The morphology and particle size distribution of the as-prepared samples were investigated using field emission scanning electron microscopy (SEM, Zeiss, ULTRA 55, German). Transmission electron microscopy (TEM, FEI, Tecnai G2 F20, USA) was used to investigate the high resolution topography and electron diffraction.

### 2.3 Electrochemical performance measurements

All electrochemical performance measurements were carried out on the electrochemical workstation (CHI760E, Shanghai Chenhua). The working electrode was prepared with TB*n* and acetylene black, which were ground and mixed uniformly. The mixture was added to an NMP solution with a PVDF content of 60% for ultrasonic dispersion to form a slurry, where the mass ratio of TB*n*, acetylene black and PVDF was 8 : 1 : 1. The slurry was uniformly coated onto the nickel current collector foam and dried at 80 °C for 12 h. The loading of TB*n* was about 2 mg cm^−2^.

The electrochemical performance of the single electrodes was evaluated by the typical three-electrode system configuration. The 6 M KOH solution served as the electrolyte, with Ag/AgCl as the reference electrode, the platinum sheet as the counter electrode, and TB*n* as the working electrode. Cyclic voltammetry (CV) curves were collected at different scan rates of 5, 10, 20, 50, 100, and 200 mV s^−1^, within a voltage window range of −1 to −0.4 V. Galvanostatic charge/discharge (GCD) curves were measured at different current densities of 0.2, 0.5, 1, 2, 5 and 10 A g^−1^. Alternating current electrochemical impedance spectroscopy (EIS) was performed between 0.01−100000 Hz at open circuit voltage with an amplitude of 5 mV.

The specific capacity (*C*_m_) values of the working electrodes determined from CV were calculated according to eqn (1), and the *C*_m_ derived from GCD was calculated according to eqn (2).1
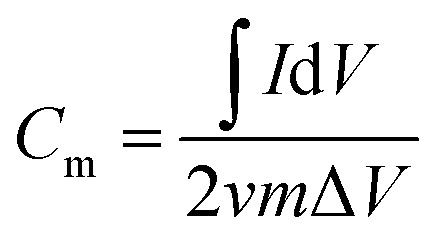
2
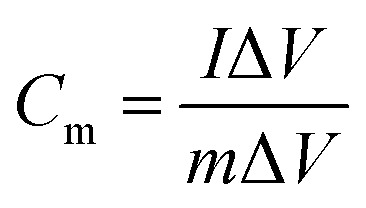
where *C*_m_ (F g^−1^) is the specific capacity, *I* (A) is the current, *v* (V s^−1^) is the potential scan rate, *m* (*g*) is the mass of active materials, Δ*V* (V) is the working potential window, and Δ*t* (s) is the discharge time.

Asymmetric supercapacitors (ASC) using the two-electrode system were used for evaluating the electrochemical performance. The outer shell consisted of a CR2032 battery case; the anode and cathode used were TB0 and TB30, respectively, with 6 M KOH as the electrolyte and a polypropylene separator. In order to balance the charge of the two electrodes, the best mass ratio of the anode and the cathode were calculated according to eqn (3).3
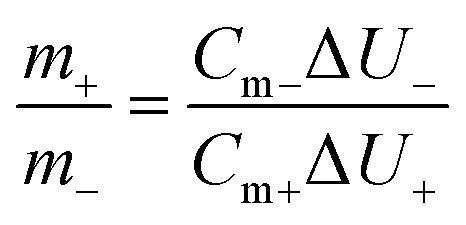
where *m*_+_ and *m*_−_ are the masses of the anode and cathode, respectively. *C*_m+_ and *C*_m−_ are the specific capacitance of the anode and cathode, respectively. Δ*U*_+_ and Δ*U*_−_ are the potential windows of the anode and cathode, respectively. The energy density and power density were calculated according to eqn (4) and (5), respectively.4
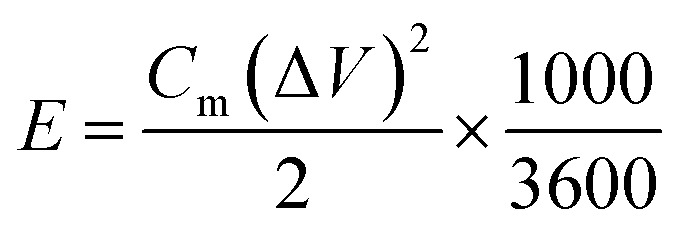
5
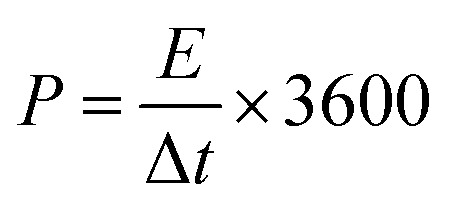
where *E* (W h kg^−1^) is the energy density, *P* (W kg^−1^) is the power density, *m* (*g*) is the mass of the active material, Δ*V* (V) is the voltage change of the discharge, Δ*t* (s) is the discharge time, and the *C*_m_ (F g^−1^) was calculated according to the eqn (2).

## 3. Results and discussion

### 3.1 Morphology and structure

**Fig. 2:**
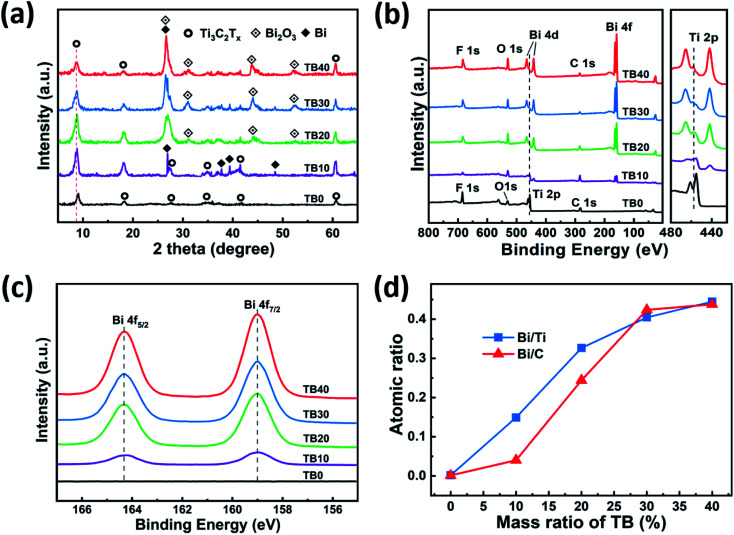
(a) XRD patterns, (b) XPS survey scan, and (c) Bi 4f high-resolution spectra of TB0−TB40. (d) The mass ratio as a function of the Bi/Ti and Bi/C atomic ratios.

Fig. 2(a) shows typical XRD patterns of TB0–TB40. The diffraction peak at 8.8° corresponds to the (002) plane of Ti_3_C_2_T_*x*_.^27^ Notably, the Ti_3_C_2_T_*x*_ (002) peaks of the TB10, TB20, TB30 and TB40 were found to gradually shift significantly to the left with the increase in Bi_2_O_3_. This result indicates that the interlayer spacing of Ti_3_C_2_T_*x*_ tends to increase due to the successful intercalation of Bi_2_O_3_ among the layers of Ti_3_C_2_T_*x*_. Diffraction peaks of Bi_2_O_3_ (ICDD#: 71-2274) were clearly visible in the spectra of TB10–TB40.^29,30^ As the Bi_2_O_3_ content increases, the diffraction peak intensity of Bi_2_O_3_ was seen to gradually increase. In addition, TB10 can be indexed to the diffraction peaks of metallic Bi (ICDD#: 05-0519),^31^ which is attributed to the carbothermal reduction of a small amount of free C atoms in Ti_3_C_2_T_*x*_ and Bi_2_O_3_ in N_2_ atmosphere during the heat treatment.^32^ Therefore, it can be inferred that the following reactions occur:6
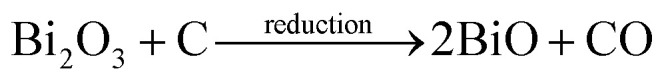
7
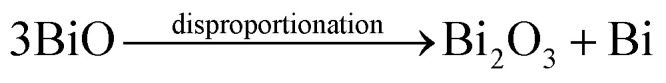


To further explore the elemental composition of all the samples, XPS analyses were then carried out for TB0–TB40. The XPS survey spectra in Fig. 2(b) show the presence of Ti, C, Bi, and O in the TB10–TB40. The Bi 4f spectra of TB0–TB40 are shown in Fig. 2(c), which exhibit binding energies at 159 eV and 164.3 eV corresponding to the characteristic peaks of Bi 4f_7/2_ and Bi 4f_5/2_, suggesting the presence of Bi_2_O_3_.^33^ The bare Bi_2_O_3_ responds to characteristic Bi 4f_7/2_ and 4f_5/2_ XPS peaks at 158.7 and 164.0 eV, respectively, acquired by the National Institute of Standards and Technology (NIST) XPS database. The negatively charged functional groups, such as O–, OH–, tend to attract electrons from bridged Bi cations that change the chemical environment and relative electronic distribution of Bi element, resulting in the peak shift of Bi 4f_7/2_ and 4f_5/2_ XPS peaks to the higher binding energy in Bi_2_O_3_–Ti_3_C_2_T_*x*_ nanocomposite.^41^ Such a phenomenon demonstrates the chemical bonding between Bi_2_O_3_ nanoparticles and Ti_3_C_2_T_*x*_ sheets in Bi_2_O_3_–Ti_3_C_2_T_*x*_ composite material. The peak intensities increase with the increasing Bi_2_O_3_–Ti_3_C_2_T_*x*_ mass ratio, from 10% to 40%, consistent with the XRD results in Fig. 2(a). The appearance of the characteristic peaks of F and O can be attributed to the corrosion of Ti_3_C_2_T_*x*_ by HF to produce a small amount of functional groups such as –F, –OH and –O.^34,35^ The C 1s and O 1s high-resolution spectra of TB0−TB40 are provided in Fig. S1 in the ESI† for comparision. Fig. S1(a)† shows the high-resolution C 1s XPS spectra of the TB0–TB40. C 1s spectrum of pristine Ti_3_C_2_T_*x*_ can be fitted into four peaks, corresponding to Ti–C–O at 282.2 eV, C–C/C–H at 284.6 eV, C–O at 286.4 eV and C=O at 288.1 eV, respectively.^19^ After the solvothermal reaction, In the spectra of the composite samples, the Ti–C–O bonds disappear and new Ti–C peaks appear at 281.2 eV. As for the O 1s XPS spectrum of pristine Ti_3_C_2_T_*x*_ in Fig. S1(b),† the peaks located at 529.8 eV, 530.4 eV, 531.2 eV, 532 eV and 533.6 eV are attributed to Ti–O, TiO_2_, C–Ti–O, C–Ti–OH and adsorbed H_2_O, respectively.^23^ The new Bi–O peaks appear at 530.1 eV and the concentration of C–Ti–OH and C–Ti–O in TB10–TB40 decreased after the solvothermal, indicating that more O combined to form Bi–O bonds. The atomic ratios of Bi, Ti and C in TB0–TB40 are shown in Fig. 2(d). The Bi/Ti and Bi/C ratios tend to increase with the increasing mass of Bi_2_O_3_, consistent with the XRD and XPS results in Fig. 2(a and b).

**Fig. 3:**
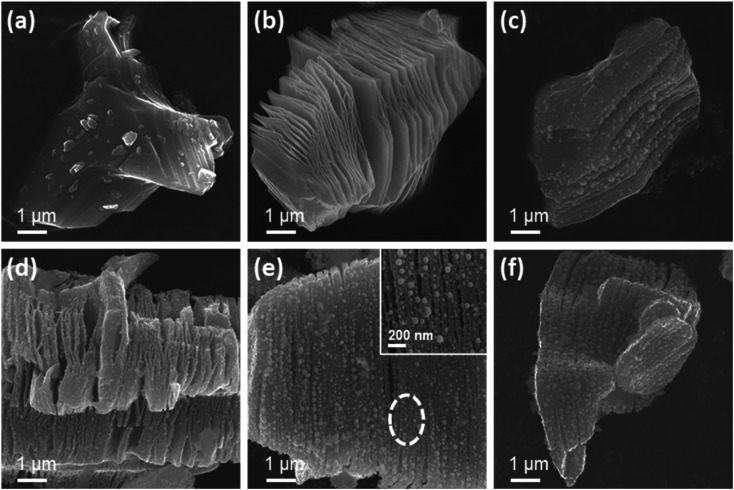
FE-SEM images of (a) Ti_3_AlC_2_, (b) TB0, (c) TB10, (d) TB20, (e) TB30, and (f) TB40.

Fig. 3 shows the morphologies of the as-prepared Ti_3_AlC_2_ and TB0–TB40. Ti_3_AlC_2_ has a ternary layered structure with a particle size of about 8 μm, as shown in Fig. 3(a). After the etching treatment to remove Al from Ti_3_AlC_2_ by HF,^36^ Ti_3_C_2_T_*x*_, with a unique accordion structure and a clean surface without impurities, was obtained, as shown in Fig. 3(b). Fig. 3(c–f) displays the structure and morphology of TB10–TB40, in which a large number of spherical Bi_2_O_3_ nanoparticles were found uniformly dispersed in the interlayer and on the surface of Ti_3_C_2_T_*x*_. A small amount of spherical Bi_2_O_3_ nanoparticles were found to have adhered on the surface of the Ti_3_C_2_T_*x*_ (TB10) in Fig. 3(c). A large number of tiny spherical Bi_2_O_3_ nanoparticles were found to be distributed between the layers of Ti_3_C_2_T_*x*_ (TB20, TB30), as shown in Fig. 3(d and e); this can effectively prevent layer stacking and collapse, exhibit higher specific surface area and improve the performance of the electric double layer capacitance.^19,37^ However, when the loading of Bi_2_O_3_ continues to increase, the surface of Ti_3_C_2_T_*x*_ (TB40) was found to be covered by numerous coarse spherical Bi_2_O_3_ nanoparticles and the lamellar structure of Ti_3_C_2_T_*x*_ was no longer obvious, as shown in Fig. 3(f).

**Fig. 4:**
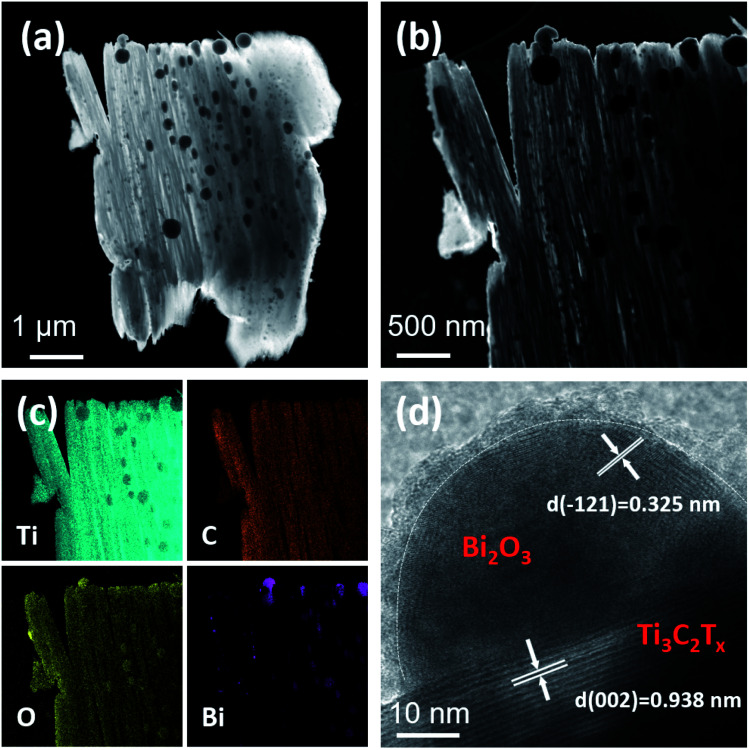
(a) TEM image, (b) HAADF-STEM image, (c) Ti, C, O, Bi elemental mapping images, and (d) HRTEM image of TB30.

To further investigate the composition and microstructure of the Bi_2_O_3_–Ti_3_C_2_T_*x*_, the representative nanocomposite, TB30, was characterized by TEM, HAADF-STEM, elemental mapping for Ti, C, O, Bi and HRTEM, as shown in Fig. 4. Fig. 4(a–c) indicates that a large number of spherical Bi_2_O_3_ nanoparticles and a small number of coarse spherical Bi_2_O_3_ nanoparticles were distributed on the Ti_3_C_2_T_*x*_. Spherical Bi_2_O_3_ nanoparticles, with an average diameter of 10–50 nm, were distributed between the layers of Ti_3_C_2_T_*x*_ and coarse spherical Bi_2_O_3_ nanoparticles, with an even particle size of around 80–200 nm, were found on the surface of Ti_3_C_2_T_*x*_. The particle-size distribution graph has been shown in Fig. S2.† The spherical Bi_2_O_3_ nanoparticles have distinct characteristics of high dispersibility and uniformity, consistent with the SEM results. Fig. 4(d) shows lattice fringe spacings of 0.325 nm and 0.938 nm, which correspond to the (−121) plane of Bi_2_O_3_ and (002) plane of Ti_3_C_2_T_*x*_,^38,39^ respectively. Significant contact was achieved between the Bi_2_O_3_ and Ti_3_C_2_T_*x*_ for more efficient electron transfer, improving the performance of electrochemistry. The spherical Bi_2_O_3_ nanoparticles were found to be covered by an amorphous Bi_2_O_3_ film with a thickness of about 2 nm, which is consistent with the findings by Wu *et al.*^24^ In addition, Fig. S3† shows a lattice fringe spacing of 0.328 nm, which corresponds to the (012) plane of metallic Bi.^40^ This proves that TB30 contains a small amount of Bi metal, which is attributed to the Bi_2_O_3_ reduced to metallic Bi by free C in Ti_3_C_2_T_*x*_, consistent with the XRD results in Fig. 1(a).

### 3.2 Electrochemical performance

**Fig. 5:**
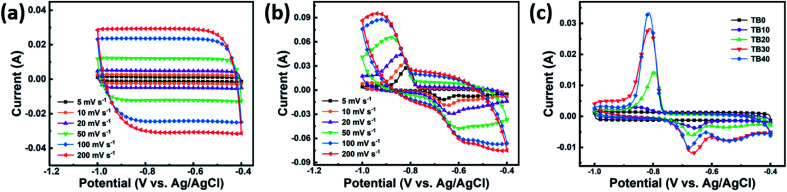
CV curves of (a) TB0 and (b) TB30 at different rates. (c) CV curves of TB0−TB40 at a scan rate of 5 mV s^−1^.

Fig. 5(a and b) show the CV curves of TB0 and TB30, respectively, at different scan rates. As shown in Fig. 5(a), the curves of TB0 look approximately like a symmetrical rectangle, which can be attributed to the double electrode layer capacitance of Ti_3_C_2_T_*x*_. All the CV curves of TB0 show a similar symmetrical rectangle with the increase in scan rate, indicating superior rate performance of TB0.^42^ The CV curves of TB30 show a prominent redox peak,^43^ which can be attributed to the introduction of Bi_2_O_3_, according to the following reactions:8
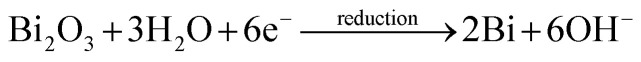
9
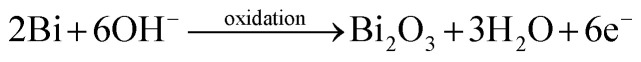


The current at the redox peak tends to slightly shift with the increase of the scan rate due to the slightly reduced conductivity resulting from the interfacial resistance between the electrode surface and the electrolyte solution, suggesting a better rate performance of TB30.^44^ The CV curves of TB0−TB40 obtained at the scan rate of 5 mV s^−1^ are shown in Fig. 5(c). It can be clearly seen that the CV curves of TB10, TB20, TB30 and TB40 show stronger redox peak currents and a larger integral than TB0, indicating the superior capacitor properties of the Bi_2_O_3_–Ti_3_C_2_T_*x*_ hybrid electrode. The best capacitance performance is obtained with TB30, *i.e.*, when the mass percentage of Bi_2_O_3_ and Ti_3_C_2_T_*x*_ is 30%.

**Fig. 6:**
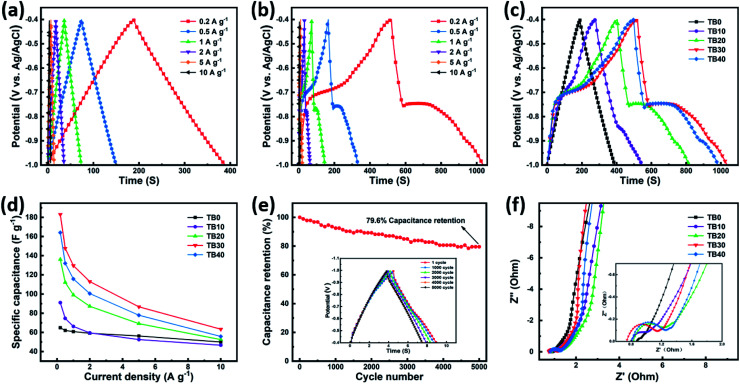
GCD curves of (a) TB0 and (b) TB30 at various current densities. (c) GCD curves of TB0−TB40 at a current density of 0.2 A g^−1^. (d) Specific capacities of TB0–TB40. (e) Cycling stability of the TB30 at a current density of 5 A g^−1^ over 5000 cycles. (f) EIS curves of TB0–TB40.

Fig. 6(a and b) show the GCD curves of TB0 and TB30, respectively, at various current densities. The GCD curves of TB0 present a typical symmetrical triangle, while those of TB30 are asymmetrical with voltage plateaus. The shape of the two curves is significantly different, which can be attributed to the pseudocapacitive characteristics of Bi_2_O_3_.^43,45^ The GCD curves of TB0–TB40 obtained at a current density of 0.2 A g^−1^ are shown in Fig. 6(c). It can be seen that the discharge time becomes longer with the increase of Bi_2_O_3_ loading. The discharge time of TB10, TB20, TB30 and TB40 was found to be longer than that of TB0, which indicates a high specific capacitance due to the presence of Bi_2_O_3_. The specific capacitance was calculated according to eqn (2); the specific capacitance of TB30 was found to be 2.8 times greater than that of TB0. In addition, the lower capacity of TB40 compared to TB30 can be ascribed to the fact that too many coarse spherical Bi_2_O_3_ nanoparticles cover the surface of Ti_3_C_2_T_*x*_, which impedes the transport of ions in the electrolyte and weakens the conductivity of Ti_3_C_2_T_*x*_.

**Fig. 7:**
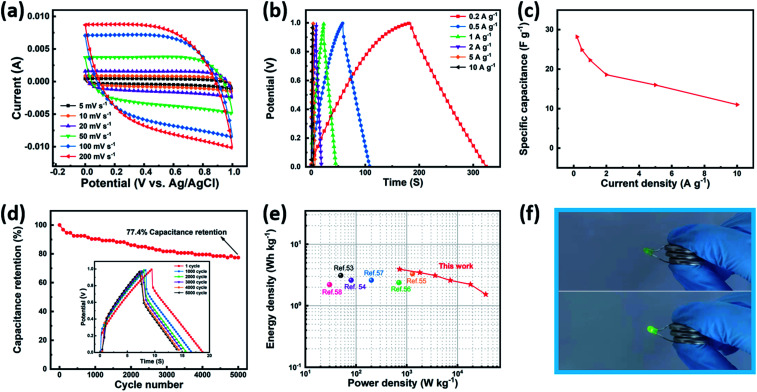
(a) CV curves of TB0//TB30 ASC device at different rates. (b) GCD curves and (c) specific capacitance of the TB0//TB30 ASC device at various current densities. (d) Cycling stability of the TB0//TB30 ASC device at a current density of 2 A g^−1^ over 5000 cycles. (e) Ragone plots of the TB0//TB30 ASC device. (f) TB0//TB30 ASC device lighting up a green LED.

Fig. 6(d) shows the variations in specific capacitance of TB0–TB40 with current density, which were calculated from the GCD curves according to eqn (2). The specific capacitance values of TB0–TB40 at a current of 0.2 A g^−1^ were found to be 64.8, 91, 136.1, 183, 164.1 F g^−1^, respectively. The specific capacitance value of the TB30 is much higher than that of all the other samples at different current densities and is attributed to a large number of tiny spherical Bi_2_O_3_ nanoparticles distributed on the surface and layers of Ti_3_C_2_T_*x*_. The spherical Bi_2_O_3_ nanoparticles tend to enlarge the spacing between the Ti_3_C_2_T_*x*_ layers, promote electron transfer, and shorten the diffusion path of ions in the electrolyte. In addition, the TB30 still exhibits a capacity of 129.7 F g^−1^ (about 70.9% retention) even at a current density of 1 A g^−1^, indicating an excellent rate capability for TB30. The specific capacitance calculated at 10 A g^−1^ is 34.6% of the specific capacitance calculated at 0.2 A g^−1^, and can be attributed to the insufficient redox reaction in the active material at high current densities, resulting in a decrease in specific capacitance.^46^ The comparison of those Ti_3_C_2_T_*x*_-based materials is shown in Table 2.^15,20,22,51^ It was clear that the performance of Bi_2_O_3_–Ti_3_C_2_T_*x*_ (TB30) electrode in this work was higher than those of reported materials. Meanwhile, as shown in Fig. 7(e), the TB30 exhibits acceptable electrochemical stability with the capacitive retention still as high as 79.6% even after 5000 cycles at 5 A g^−1^, and can be attributed to the expansion and contraction of Bi_2_O_3_ during the insertion and embedding of electrolyte ions by charging and discharging, resulting in a decrease in the capacity.

**Table 2:** Comparison of capacitance performance of Ti_3_C_2_T_*x*_-based materials, where *C*_m_ is specific capacitance

Materials	Electrolyte	*C*_m_	Rate	Ref.
Bi_2_O_3_–Ti_3_C_2_T_*x*_	KOH	183 F g^−1^	0.2 A g^−1^	This work
Ti_3_C_2_T_*x*_	KOH	∼88 F g^−1^	5 mV s^−1^	15
TiO_2_–Ti_3_C_2_T_*x*_	KOH	143 F g^−1^	5 mV s^−1^	20
CNT-d-Ti_3_C_2_T_*x*_	MgSO_4_	150 F g^−1^	2 mV s^−1^	22
MoO_3_–Ti_3_C_2_T_*x*_	KOH	150.7 F g^−1^	2 mV s^−1^	51

Nyquist impedance plots of TB0–TB40 are shown in Fig. 6(f). The equivalent series resistance (ESR) of electrodes, which is related to the ionic resistance of the electrolyte, the internal resistance of the active material and the current collector, and the interface contact resistance between the electrode and the electrolyte impedance of the electrolyte,^47^ depends on the intercept of the semicircle at the *X*-axis in the high frequency range. It can be seen that the ESR values of TB0–TB40 are 0.87, 0.79, 0.82, 0.73 and 0.80 Ω, respectively. Among them, TB30 has the smallest ESR. The charge transfer resistance (*R*_ct_) depends on the arc diameter of the semicircle.^48^ The *R*_ct_ values of TB10, TB20, TB30 and TB40 are much larger than that of TB0, which can be attributed to the poor conductivity of Bi_2_O_3_. Simultaneously, the increase of resistance reveals the successful incorporation of Bi_2_O_3_. The linear region in the low frequency range is related to the diffusion resistance of the ions in the electrolyte.^49^ The linear part of TB30 is closest to the vertical, indicating that the electrolyte ion diffusion has the lowest resistance. The above results prove that TB30 has good electrochemical capacitance behavior, consistent with the results shown in Fig. 6(d).

In order to further study the Bi_2_O_3_–Ti_3_C_2_T_*x*_ nanocomposite for practical applications, an asymmetric supercapacitor (ASC) was constructed,^50^ with TB0 and TB30 as the positive and negative electrodes, respectively. Fig. 7(a) shows the CV curves of the TB0//TB30 ASC at different scan rates. It can be seen that the shape of the CV curves of the TB0//TB30 ASC remained the same at different scan rates, implying excellent rate capacity and good reversibility, indicating the good synergistic effect of the electrochemical behavior of both TB0 and TB30 electrodes. The GCD curves of TB0//TB30 ASC at various current densities are shown in Fig. 7(b). The non-linearity of the curve is due to the redox reaction that occurred during the charging and discharging process. The specific capacitance of the TB0//TB30 ASC at different current densities in Fig. 7(c), indicates that the capacitive retention is still as high as 79.1% even after the current density is increased from 0.2 A g^−1^ to 1 A g^−1^, and the specific capacitance is reduced from 28.2 F g^−1^ to 22.3 F g^−1^. In addition, the specific capacitance calculated at 10 A g^−1^ is 11 F g^−1^ with a capacitive retention of 39%, which is attributed to the insufficient redox reaction in the active material at high current density, resulting in a decrease in specific capacitance. This is consistent with the trend of the capacitive retention of TB30. Meanwhile, as shown in Fig. 7(d), the TB0//TB30 ASC exhibits acceptable electrochemical stability with the capacitive retention still as high as 77.4% even after 5000 cycles at 2 A g^−1^ and higher than those of reported devices such as Ni(OH)_2_-CNT//Bi–Bi_2_O_3_-CNT based ASC (72.9%/1000 cycles at 1 A g^−1^),^24^ PPy-Ti_3_C_2_T_*x*_//PPy-Ti_3_C_2_T_*x*_ based SC (73.68%/4000 cycles at 1 A g^−1^).^52^ To further illustrate the overall electrochemical characteristics of the TB0//TB30 ASC, a Ragone plot of the TB0//TB30 ASC was carried out and compared with that of other reported devices based on the a two-electrode configuration in Fig. 7(d) and Table 3.^53–58^ The highest energy density of the TB0//TB30 ASC is 3.92 W h kg^−1^ at a power density of 720 W kg^−1^, and the highest power density of the TB0//TB30 ASC is 36 000 W kg^−1^ at an energy density of 1.53 W h kg^−1^. The energy density and power density of our device is higher than that of many reported Ti_3_C_2_T_*x*_-based and carbon-based SC and ASC devices. The Fig. 7(f) shows that three TB0//TB30 ASC coin cells in series could power a green light-emitting diode (LED, 3 V, 20 mA). Based on above mentioned results, it can be conclued that Bi_2_O_3_–Ti_3_C_2_T_*x*_ is a promising electrode material for supercapacitors in the future.

**Table 3:** Comparison of the performances of the various electrochemical supercapacitors

Supercapacitors	*P* (W kg^−1^)	*E* (W h kg^−1^)	Ref.
TB0//TB30	720	3.92	This work
RGO//RGO	50	3.1	53
FC//MnO-RGO	80	2.6	54
Ti_3_C_2_T_*x*_//CFP	1300	3.3	55
N–Ti_3_C_2_T_*x*_//N–Ti_3_C_2_T_*x*_	700	2.4	56
PANI@CNT//PANI@CNT	200	2.6	57
Ti_3_C_2_T_*x*_//Ti_3_C_2_T_*x*_	30	2.2	58

## 4. Conclusions

In this study, we prepared Bi_2_O_3_–Ti_3_C_2_T_*x*_ nanocomposites by a solvothermal method. The spherical Bi_2_O_3_ nanoparticles were uniformly distributed on the surface and layers of Ti_3_C_2_T_*x*_, which effectively prevented layer stacking and increased the inter-layer spacing. When the mass percentage of Bi_2_O_3_ and Ti_3_C_2_T_*x*_ was 30% (TB30), the best capacitance performance was obtained. The specific capacitance of TB30 was 2.8 times that of TB0 at 0.2 A g^−1^, which was attributed to the outstanding pseudocapacitance of Bi_2_O_3_ and the improvement of layer stacking of Ti_3_C_2_T_*x*_. However, too many coarse spherical Bi_2_O_3_ nanoparticles tend to cover the surface of Ti_3_C_2_T_*x*_ (TB40), impeding the transport of ions in the electrolyte and weakening the electrochemical performance. The asymmetric supercapacitor device assembled with TB0 as the positive electrode and TB30 as the negative electrode displayed a high energy density of 3.92 W h kg^−1^ and a maximum power density of 36 000 W kg^−1^, and maintained 77.4% of the initial capacitance after 5000 cycles at a current density of 2 A g^−1^. This work shows that Bi_2_O_3_–Ti_3_C_2_T_*x*_ as a negative material has excellent prospects in high-performance electrochemical energy storage devices like supercapacitors.

## Conflicts of interest

There are no conflicts to declare.

## Supplementary Material

NA-003-D1NA00443C-s001

## References

[cit1] Gonzalez A., Goikolea E., Barrena J. A., Mysyk R. (2016). Renewable Sustainable Energy Rev..

[cit2] Afir A., Rahman S. M. H., Azad A. T., Zaini J., Islan M. A., Azad A. K. (2019). Journal of Energy Storage.

[cit3] Liu Y., Wang Z., Zhong Y., Xu X., Veder J. M., Rowles M. R., Saunders M., Ran R., Shao Z. (2020). Chem. Eng. J..

[cit4] Liu Y., Jiang S. P., Shao Z. (2020). Mater. Today Adv..

[cit5] Chen L. (2017). Energy Environ. Sci..

[cit6] Liu C., Wang J., Li J., Zeng M., Luo R., Shen J., Sun X., Han W., Wang L. (2016). ACS Appl. Mater. Interfaces.

[cit7] Shao Y., El-Kady M. F., Sun J., Li Y., Zhang Q., Zhu M., Wang H., Dunn B., Kaner R. B. (2018). Chem. Rev..

[cit8] Wang Z., Xu Z., Huang H., Chu X., Xie Y., Xiong D., Yan C., Zhao H., Zhang H., Yang W. (2020). ACS Nano.

[cit9] Yi C., Zou J., Yang H., Leng X. (2018). Trans. Nonferrous Met. Soc. China.

[cit10] Zhang L., Huang D., Hu N., Yang C., Li M., Wei H., Yang Z., Su Y., Zhang Y. (2017). J. Power Sources.

[cit11] Shen B., Zhang X., Guo R., Lang J., Chen J., Yan X. (2016). J. Mater. Chem. A.

[cit12] Lin Z., Sun D., Huang Q., Yang J., Barsoum M. W., Yan X. (2015). J. Mater. Chem. A.

[cit13] Beguin F., Presser V., Balducci A., Frackowiak E. (2014). Adv. Mater..

[cit14] Naguib M., Kurtoglu M., Presser V., Lu J., Niu J., Heon M., Hultman L., Gogotsi Y., Barsoum M. W. (2011). Adv. Mater..

[cit15] Tang Y., Zhu J., Yang C., Wang F. (2016). J. Electrochem. Soc..

[cit16] Su C., Liu Y., Luo Z., Veder J., Zhong Y., Jiang S. P., Shao Z. (2021). Chem. Eng. J..

[cit17] Lukatskaya M. R., Bak S., Yu X., Yang X., Barsoum M. W., Gogotsi Y. (2015). Adv. Energy Mater..

[cit18] Li H., Hou Y., Wang F., Lohe M. R., Zhuang X., Niu L., Feng X. (2017). Adv. Energy Mater..

[cit19] Yuan W., Cheng L., Zhang B., Wu H. (2018). Ceramurgia Int..

[cit20] Zhu J., Tang Y., Yang C., Wang F., Cao M. (2016). J. Electrochem. Soc..

[cit21] Zhu M., Huang Y., Deng Q., Zhou J., Pei Z., Xue Q., Huang Y., Wang Z., Li H., Huang Q., Zhi C. (2016). Adv. Energy Mater..

[cit22] Zhao M., Ren C. E., Ling Z., Lukatskaya M. R., Zhang C., Van Aken K. L., Barsoum M. W., Gogotsi Y. (2015). Adv. Mater..

[cit23] Yang S., Qian L., Ping Y., Zhang H., Li J., Xiong B., Fang P., He C. (2021). Ceramurgia Int..

[cit24] Wu H., Guo J., Yang D. A. (2020). J. Mater. Sci. Technol..

[cit25] Deepi A., Srikesh G., Nesaraj A. S. (2018). Nano-Struct. Nano-Objects.

[cit26] Shaikh Z. A., Shinde P. V., Shaikh S. F., Al-Enizi A. M., Mane R. S. (2020). Solid State Sci..

[cit27] Feng A., Yu Y., Wang Y., Jiang F., Yu Y., Le M., Song L. (2017). Mater. Des..

[cit28] Mashtalir O., Lukatskaya M. R., Kolesnikov A. I., Raymundo-Piñero E., Naguib M., Barsoum M. W., Gogotsi Y. (2016). Nanoscale.

[cit29] Sood S., Umar A., Kumar Mehta S., Kumar Kansal S. (2015). Ceramurgia Int..

[cit30] Mishchenko K. V., Gerasimov K. B., Yukhin Y. M. (2020). Mater. Today: Proc..

[cit31] Qu L., Luo Z., Tang C. (2013). Mater. Res. Bull..

[cit32] Chen W., Pan X., Bao X. (2007). J. Am. Chem. Soc..

[cit33] Liu R., Ma L., Niu G., Li X., Li E., Bai Y., Yuan G. (2017). Adv. Funct. Mater..

[cit34] Naguib M., Mashtalir O., Carle J., Presser V., Lu J., Hultman L., Gogotsi Y., Barsoum M. W. (2012). ACS Nano.

[cit35] Chang F., Li C., Yang J., Tang H., Xue M. (2013). Mater. Lett..

[cit36] Wang F., Yang C., Duan C., Xiao D., Tang Y., Zhu J. (2014). J. Electrochem. Soc..

[cit37] Xu H., Zheng D., Liu F., Li W., Lin J. (2020). J. Mater. Chem. A.

[cit38] Qin F., Zhao H., Li G., Yang H., Li J., Wang R., Liu Y., Hu J., Sun H., Chen R. (2014). Nanoscale.

[cit39] Feng W., Luo H., Zeng S., Chen C., Deng L., Tan Y., Zhou X., Peng S., Zhang H. (2018). Mater. Chem. Front..

[cit40] Chen M., Li Y., Wang Z., Gao Y., Huang Y., Cao J., Ho W., Lee S. (2017). Ind. Eng. Chem. Res..

[cit41] Deng Z., Liu T., Chen T., Jiang J., Yang W., Guo J., Zhao J., Wang H., Gao L. (2017). ACS Appl. Mater. Interfaces.

[cit42] Zhang M., Chen X., Sui J., Abraha B. S., Li Y., Peng W., Zhang G., Zhang F., Fan X. (2020). Inorg. Chem. Front..

[cit43] Vivier V., Regis A., Sagon G., Nedelec J. Y., Yu L. T., Cachet-Vivier C. (2001). Electrochim. Acta.

[cit44] Wu Q., Xu Y., Yao Z., Liu A., Shi G. (2010). ACS Nano.

[cit45] Yang S., Qian L., Ping Y., Zhang H., Li J., Xiong B., Fang P., He C. (2021). Ceramurgia Int..

[cit46] Pan Z., Cao F., Hu X., Ji X. (2019). J. Mater. Chem. A.

[cit47] Zhang X., Liu X., Yan R., Yang J., Liu Y., Dong S. (2020). J. Mater. Chem. C.

[cit48] Tang H., Wang J., Yin H., Zhao H., Wang D., Tang Z. (2015). Adv. Mater..

[cit49] Ahmed B., Anjum D. H., Gogotsi Y., Alshareef H. N. (2017). Nano Energy.

[cit50] Venkateshalu S., Grace A. N. (2020). Electrochim. Acta.

[cit51] Antiohos D., Pingmuang K., Romano M. S., Beirne S., Romeo T., Aitchison P., Minett A., Wallace G., Phanichphant S., Chen J. (2013). Electrochim. Acta.

[cit52] Wei D., Wu W., Zhu J., Wang C., Zhao C., Wang L. (2020). J. Electroanal. Chem..

[cit53] Wang Z., Xu Z., Huang H., Chu X., Xie Y., Xiong D., Yan C., Zhao H., Zhang H., Yang W. (2020). ACS Nano.

[cit54] Liu Y., Wang Z., Zhong Y., Xu X., Veder J. M., Rowles M. R., Saunders M., Ran R., Shao Z. (2020). Chem. Eng. J..

[cit55] Liu Y., Jiang S. P., Shao Z. (2020). Mater. Today Adv..

[cit56] Navarro-Suárez A. M., Van Aken K. L., Mathis T., Makaryan T., Yan J., Carretero-González J., Rojo T., Gogotsi Y. (2018). Electrochim. Acta.

[cit57] Wu W., Lin S., Chen T., Li L., Pan Y., Zhang M., Wu L., Gao H., Zhang X. (2017). J. Alloys Compd..

[cit58] Tang Y., Zhu J., Yang C., Wang F. (2016). J. Electrochem. Soc..

