# Supplementary material for: Solvothermal preparation of spherical Bi2O3 nanoparticles uniformly distributed on Ti3C2Tx for enhanced capacitive performance
Source: Nanoscale Adv. 2021 Aug 5;3(18):5312–21. doi: 10.1039/d1na00443c (PMC9417557; doi:10.1039/d1na00443c)
Supplement: NA-003-D1NA00443C-s001 [file NA-003-D1NA00443C-s001.pdf]

Electronic Supplementary Material (ESI) for Nanoscale Advances.
This journal is © The Royal Society of Chemistry 2021

Supporting Information

Solvothermal Preparation of Spherical Bi2O3 Nanoparticles Uniformly Distributed on Ti3C2Tx for Enhanced Capacitive Performance

Tao Li,[a,b] *Xuefeng Chang,*[a,b] *Lifang Mei,*[a,b] *Xiayun Shu,**[a,b] *Jidong Ma,*[c] *Li Ouyang,*[a,b] *Siyong Gu,**[c]

[a] *Mechanical and Automotive Engineering, Xiamen University of Technology, Fujian, Xiamen, 361024. P. R. China.*

[b] *Institute of Precision Actuation and Transmission, Fujian, Xiamen, 361024. P. R. China.*

[c] *The Key Laboratory for Power Metallurgy Technology and Advanced Materials of Xiamen, Fujian, Xiamen, 361024. P. R. China.*

***Corresponding author** *E-mail address: shuxiayun@xmut.edu.cn (Xiayun Shu) gu-siyong@163.com (Siyong Gu),*

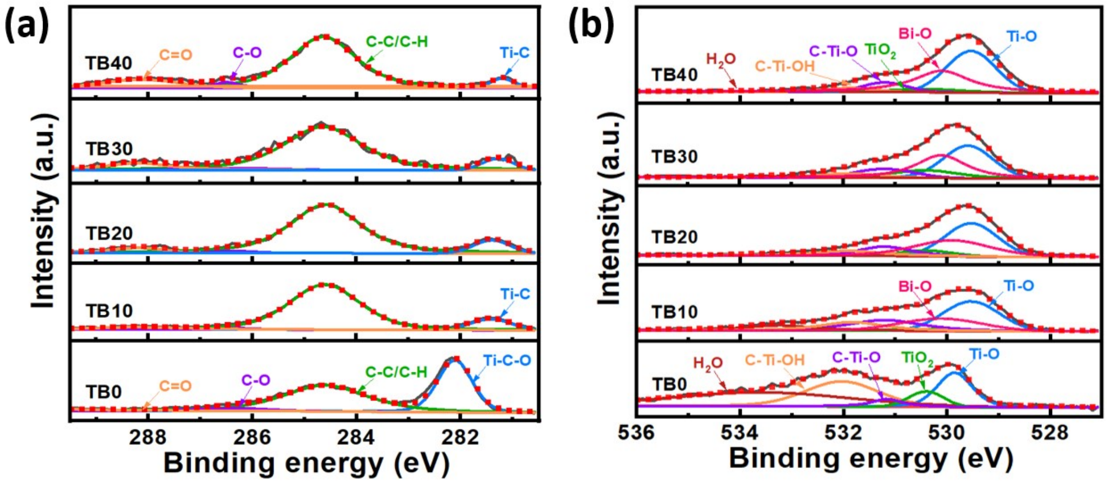

Fig. S1 (a) C 1s and (b) O 1s high-resolution spectra of TB0−TB40

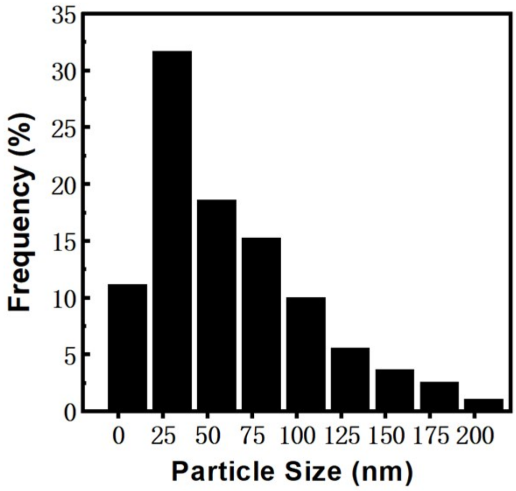

Fig. S2 Particle-size distribution of TB30

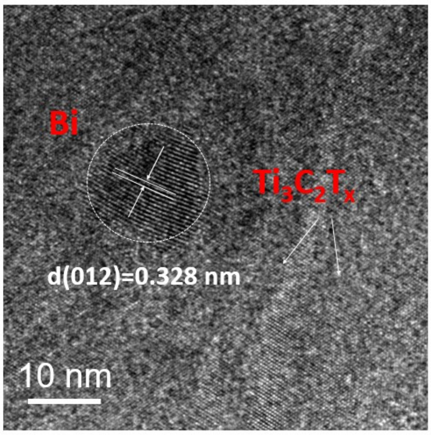

Fig. S3 HRTEM images of TB30.
